# Supplementary material for: Drug repurposing for aging research using model organisms
Source: Aging Cell. 2017 Jun 16;16(5):1006–15. doi: 10.1111/acel.12626 (PMC5595691; doi:10.1111/acel.12626)
Supplement: Supplementary file 7 — Data S1 Zip‐Archive of all report cards. [file ACEL-16-1006-s007.zip › RC_PGO.pdf]

PGO

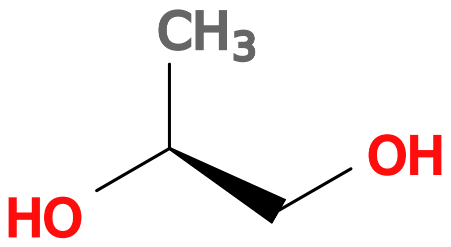

Database identifiers

CHEBI	29002
ZINC	ZINC00895316
eMolecules	490838

Ranking

	Rank	Score
Drosophila	NA	NA
C. elegans	303/591	0.19

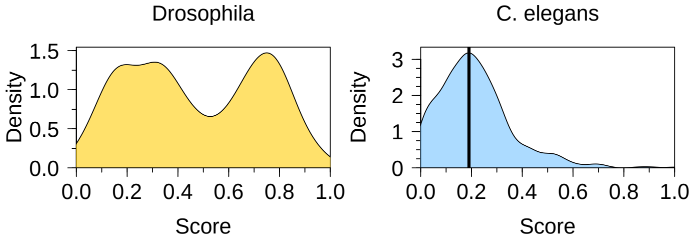

	Ageing implication	Domain conservation	Binding site conservation	Binding affinity	Bioavailability	Lipinski	Promiscuity	Purchasability	Drug approval	**Total**
Drosophila	NA	NA	NA	NA	NA	NA	NA	NA	NA	NA
C. elegans	0.624	0.666	0.612	0.291	0.211	0.0	-0.0	0.1	0.075	0.19

Names

- **(S)-propane-1,2-diol**
- (S)-1,2-Propanediol
- (S)-Propylene glycol

Roles

human metabolite

Status

Number of Rule of 5 violations	0
Binding affinity to original target in log units (RF-Score prediction)	4.11
Burns *C. elegans* bioavailability prediction	-5.56

Compound Target Characteristics

Matrix metalloproteinase-9

Best gene implication in ageing for this target family came from gene D3ZYK8 via mapping the annotation from RGD 621320 annotated in RGD 2014-03-11. Annotation GO 7568 (aging) was Inferred from Expression Pattern

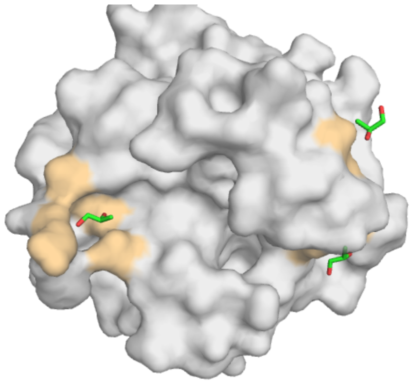

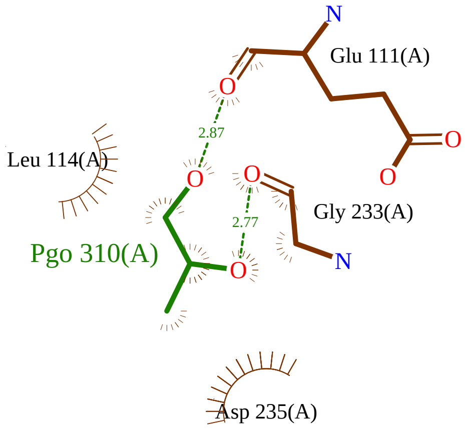

protein	amino acids contacts (binding site)
PDB:4h3x:chainA:P14780	E L D V A E D D E L G Y G D
PDB:4hma:chainA:P14780	E L D V A E D D E L G Y G D
PDB:4jij:chainA:P14780	E L D V A E D D E L G Y G D
PDB:4jqg:chainA:P14780	E L D V A E D D E L G Y G D
sp:P14780:MMP9_HUMAN	E L D V A E D D E L G Y G D
tr:D3ZYK8:D3ZYK8_RAT	E L D V A E D D E L G Y G D
sp:P41245:MMP9_MOUSE	K L D V A E D D E L G Y G D
tr:G5EBU3:G5EBU3_CAEEL	S F D V R N D D E N A T G E

	whole protein		domain-based		contact-based	
protein	ident	simil	ident	simil	ident	simil
PDB:4h3x:chainA:P14780	0.99	0.99	0.99	0.99	1.0	1.0
PDB:4hma:chainA:P14780	0.99	1.0	0.99	1.0	1.0	1.0
PDB:4jij:chainA:P14780	0.98	0.99	0.98	0.99	1.0	1.0
PDB:4jqg:chainA:P14780	0.98	0.99	0.98	0.99	1.0	1.0
sp:P14780:MMP9_HUMAN	1.0	1.0	1.0	1.0	1.0	1.0
tr:D3ZYK8:D3ZYK8_RAT	0.75	0.92	0.84	0.95	1.0	1.0
sp:P41245:MMP9_MOUSE	0.72	0.89	0.82	0.94	0.93	0.96
tr:G5EBU3:G5EBU3_CAEEL	0.18	0.45	0.33	0.67	0.43	0.61
